# Enhanced Roles of Carbon Architectures in High-Performance Lithium-Ion Batteries

**DOI:** 10.1007/s40820-018-0233-1

**Published:** 2019-01-10

**Authors:** Lu Wang, Junwei Han, Debin Kong, Ying Tao, Quan-Hong Yang

**Affiliations:** 1 Nanoyang Group, State Key Laboratory of Chemical Engineering, School of Chemical Engineering and Technology, Collaborative Innovation Center of Chemical Science and Engineering (Tianjin), Tianjin University, Tianjin, 300072 People’s Republic of China; 2 CAS Key Laboratory of Nanosystem and Hierarchical Fabrication, CAS Center for Excellence in Nanoscience, National Center for Nanoscience and Technology, Beijing, 100190 People’s Republic of China

**Keywords:** Lithium-ion battery, Carbon architecture, Energy density, Power density, Assembly

## Abstract

Lithium-ion batteries (LIBs), which are high-energy-density and low-safety-risk secondary batteries, are underpinned to the rise in electrochemical energy storage devices that satisfy the urgent demands of the global energy storage market. With the aim of achieving high energy density and fast-charging performance, the exploitation of simple and low-cost approaches for the production of high capacity, high density, high mass loading, and kinetically ion-accessible electrodes that maximize charge storage and transport in LIBs, is a critical need. Toward the construction of high-performance electrodes, carbons are promisingly used in the enhanced roles of active materials, electrochemical reaction frameworks for high-capacity noncarbons, and lightweight current collectors. Here, we review recent advances in the carbon engineering of electrodes for excellent electrochemical performance and structural stability, which is enabled by assembled carbon architectures that guarantee sufficient charge delivery and volume fluctuation buffering inside the electrode during cycling. Some specific feasible assembly methods, synergism between structural design components of carbon assemblies, and electrochemical performance enhancement are highlighted. The precise design of carbon cages by the assembly of graphene units is potentially useful for the controlled preparation of high-capacity carbon-caged noncarbon anodes with volumetric capacities over 2100 mAh cm^−3^. Finally, insights are given on the prospects and challenges for designing carbon architectures for practical LIBs that simultaneously provide high energy densities (both gravimetric and volumetric) and high rate performance.

## Highlights

- Assembly strategies that reinforce the roles of carbon architectures as active materials, electrochemical reaction frameworks, and current collectors in high-energy and high-power lithium-ion batteries are summarized.
- To enhance structural stability and volumetric performance, the rational design of carbon architectures for high-capacity noncarbons in terms of the interface, network skeleton, void space, and densification, is discussed in detail.
- Designing carbon cages that protect the electroactive noncarbon is highlighted as a promising strategy that solves the challenges associated with future high-capacity noncarbon anode construction.

## Graphical abstract

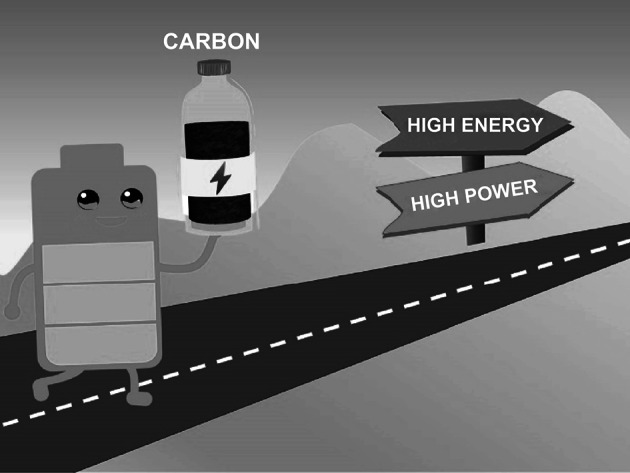

## Introduction

Developing rechargeable electrochemical energy storage (EES) devices represents one of the most promising approaches to achieving high-performance energy storage, since they can provide large-scale and smart-grid energy storage with high levels of efficiency [1–5]. Over the past two decades, lithium-ion batteries (LIBs) have played key roles as EES devices in electronics applications, electrical vehicles, and large-grid energy systems due to their high energy densities and low safety risks [6–9]. Efforts have been made to engineer current graphitic anodes and transition metal oxide cathodes with enhanced ion diffusion and electrical conductivity for high-energy and high-power LIBs. Nevertheless, the further development of LIBs is largely constrained by the low theoretical capacities (100–350 mAh g^−1^) of graphitic anodes and metal oxide cathodes based on the intercalation mechanism [10]. To further improve the energy densities of LIBs, noncarbon anodes that operate beyond the intercalation mechanism have emerged as promising alternatives for conventional graphitic anodes due to their higher theoretical specific capacities (1000–4000 mAh g^−1^). However, high-capacity noncarbon anodes suffer from drastic volume changes during cycling, which largely block their structural stabilities and induce repetitive electrolyte decomposition, leading to low Coulombic efficiency and rapid capacity fading. Even worse, a high-density and high-mass-loaded electrode that offers high energy density usually suffers from poor rate performance and structural failure owing to the large tortuosity associated with charge-carrier transport and accumulated volume expansion stress [11, 12]. Therefore, to improve its energy density and further ensure its high-power capability and long cycle life, the electrode should be well designed to accommodate more charge, improve charge transport, and further enhance the structural stability of the electrode in terms of its density and thickness.

To improve energy and power densities, carbons have been widely used in LIBs, including, but not limited to, their use as active materials, conductive additives, and electrochemical reaction frameworks in electrodes. Graphite, as the-state-of-the-art anode material, was the final finishing touch for the commercialization of LIBs in 1991 [13]. Carbons are also promisingly introduced in high-capacity noncarbon active materials to buffer volume fluctuations and improve electrical conductivity, in order to build better LIBs. Highly conductive *sp*^2^-type carbons are used as effective conductive additives to improve the electron transport in low-conductivity transition metal compound cathodes. Notably, in advanced carbon-based electrode research, the development of carbon nanostructures has been demonstrated as an alternative approach to improving the electrochemical performance and extending the cycle lives of LIBs, on account of their unique structural features, such as high specific surface areas, large amounts of pores, and short ion- and electron-transport pathways for electroactive particles [14]. Unfortunately, issues associated with the low tap density, low Coulombic efficiency, and prolonged charge-transfer distance across the electrode have also accompanied the introduction of carbon nanomaterials in electrodes, which blocks the electrochemical performance of electrodes based on practical packing densities and thicknesses, thereby hindering the ability to achieve high energy and power densities in LIBs [15]. Hence, assembly strategies have been developed that realize synergies between primary carbon nanostructures and advanced carbon architectures in high-performance electrodes. Different dimensional carbon nanomaterials, including zero-dimensional (0D) nanoparticles [16], one-dimensional (1D) nanowires or nanorods [17, 18], and two-dimensional (2D) nanoflakes [19, 20], etc., as building blocks, have been used to construct rational carbon architectures through suitable assembly methods and controllable experimental conditions. The assembly of these carbon-based nanomaterials not only retains their intrinsic electrical and mechanical properties, but also generates new characteristics through the achievement of close connections between the noncarbon units, interpenetrated charge-transport networks, large electrochemical reaction surface areas, intact electrolyte blocking layers, and high packing densities.

**Fig. 1:**
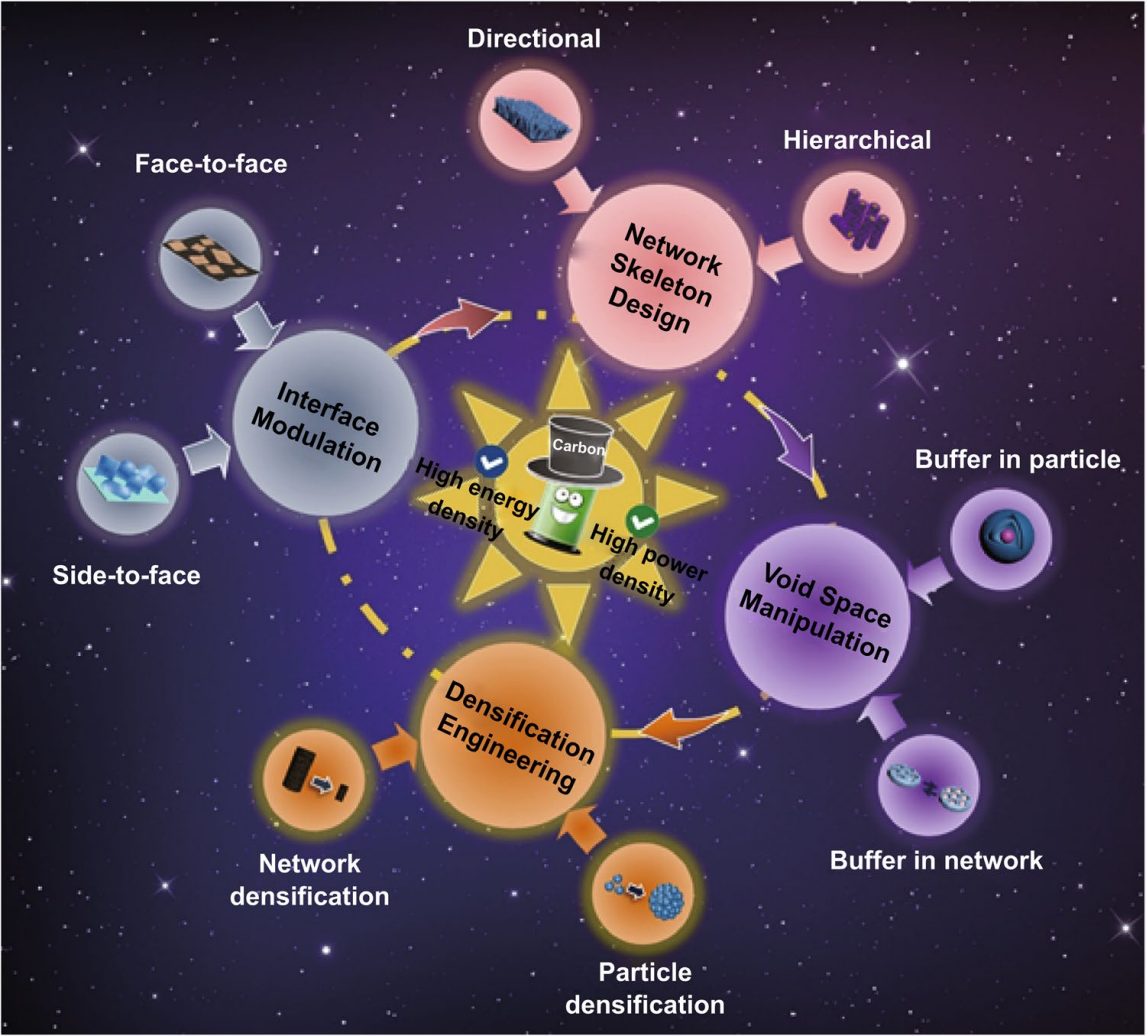
Carbon-architecture assembly strategies for building high-energy and high-power LIBs

A number of recent reviews have summarized the critical roles of carbon materials, such as carbon nanotubes, graphene, and carbon composites, in EES devices [21–23]. Fang et al. offered a general and objective understanding of the roles of carbon nanotubes and graphene in regulating lithium storage process in electroactive materials [21]. Our group discussed the use of graphene as soft templates in the design of well-controlled carbon composites for enhanced electrochemical performance [22]. In this review, we focus on recent research highlights of assembly strategies that reinforce the roles of the carbon architecture toward achieving high-energy and high-power LIBs (Fig. 1). First, we briefly discuss assembly methods used for carbon-based nanomaterials that enable the controllable construction of various morphologies and internal structures. Then, assembly strategies used for the construction of carbon architectures with enhanced roles of active materials, electrochemical reaction frameworks, and current collectors, which solve specific issues in electrodes, including low electronic conductivities, poor ion diffusions, low densities, side reactions with electrolytes, and large volume changes, will be discussed in detail. Strategies for designing carbon architectures, such as interfacial modulation, network skeleton design, void space manipulation, and densification engineering, are highlighted. In particular, the significance of the carbon-cage design for noncarbon materials is demonstrated by superior volumetric lithium storage and stable electrode structures during cycling. Finally, a brief summary and some perspectives on the applications of carbon architectures in practical LIBs are offered.

## Assembly Methods for the Construction of Carbon Architectures

**Fig. 2:**
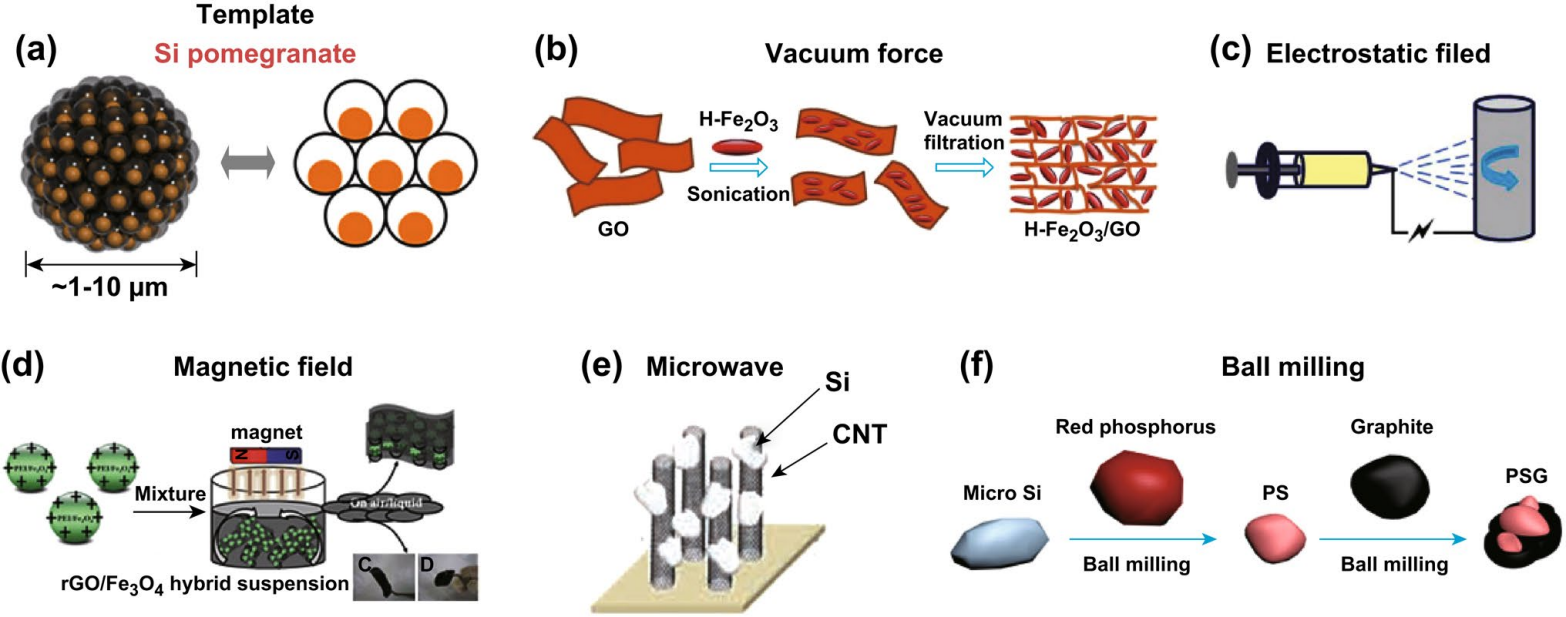
Assembly methods with the assistance of external forces or fields. **a** Schematic of silicon pomegranates. Reprinted with permission from Ref. [30]. **b** Schematic of the fabrication process for H-Fe_3_O_4_/GS hybrid films. Reprinted with permission from Ref. [39]. **c** Schematic of the gradient electrospinning and controlled pyrolysis method. Reprinted with permission from Ref. [45]. **d** Schematic of the synthesis rGO/Fe_3_O_4_ hybrid paper. Reprinted with permission from Ref. [52]. **e** VA-CNTs/Si structure. Reprinted with permission from Ref. [56]. **f** Schematic of the fabrication processes for nanostructured P-doped Si/graphite composites. Reprinted with permission from Ref. [57]

The fabrication of carbon-based materials with rationally designed structures and properties that directly determine their potentials in EES applications is of crucial importance. Assembling carbon nanostructures into architectures not only retains the intrinsic properties of the carbon building blocks, but also facilitates good control over nanostructural arrangements and allows properties to be manipulated. Typical assembly methods, including electrospinning [24], chemical vapor deposition (CVD) [25], and self-assembly [26], have been widely used to synthesize carbon architectures. To further enhance controllability during carbon assembly, external influences, such as template assistance, vacuum force, electric- and magnetic field guidance, have been widely introduced and well developed in recent years. Hence, the following part mainly focuses on external force control during the carbon assembly process (Fig. 2).

Template-assisted assembly is one of the most frequently used strategies for fabricating hierarchically structured carbon-based materials. During the fabrication processes, the introduction of templates makes it easy to precisely control the sizes, shapes, structures, and properties of carbon-based nanomaterials for producing specially structured materials, such as nanofibers [27], nanotube arrays [28], core–shell structures [29], pomegranate-like structures [30], and 3D composite aerogels [31]. For example, with the assistance of porous alumina templates, coaxial manganese oxide/carbon nanotube (MnO_2_/CNT) arrays were fabricated by a combination of simple vacuum infiltration and CVD techniques, followed by alkali treatment to remove the templates [28]. Nevertheless, conventional templating methods may result in structural collapse during template-removal treatments, making it difficult to obtain more complicated structures (e.g., yolk-shelled and multi-shelled structures). Hence, a few advanced sacrificial template approaches, such as self-templating [32–34], chemical oxidation [35], and thermal decomposition [36] methods, have been well developed. Typically, self-templated strategies can directly convert templates into hollow structures by controlled etching, outward diffusion, or heterogeneous contraction [37]. For instance, using a metal–organic framework (ZIF-67) as a self-template, Xia et al. synthesized a cobalt-phosphide-based (Co_x_P-NC) nanohybrid in situ [33], in which Co_x_P nanoparticles were homogeneously embedded in a polyhedral porous nitrogen-doped carbon, which not only contributed to fast electron and lithium-ion transport, but also provided sufficient buffer space.

Force caused by pressure difference is another commonly used external driving force in material preparation, especially in the vacuum filtration method. The pressure difference between the two sides of the filter medium drives the deposition of the solid matter in the suspension onto the filter medium, which then forms a uniform film or paper-supported electrode, such as a cellulose-based paper anode [38], a hollow ferroferric oxide/graphene (H–Fe_3_O_4_/GS) hybrid film [39], or a silicon/Cladophora nanocellulose/carbon nanotube hybrid film [40]. For example, using a simple filtration method, free-standing porous silicon nanowire (Si-NW) and graphene nanoribbon (GNR) papers were obtained, which possessed homogenous conductive pathways [41]. However, the difference in the filtration rates of various components in the suspension may result in an uneven distribution of components in the membrane. To solve the above problems, Gao et al. adopted a coupling technique involving spray deposition and vacuum filtration to produce a high-density (2.7 mg cm_electrode_^−3^) and high-mass-loading (LiFePO_4_ loading of 5 mg cm^−2^) lithium iron phosphate (LiFePO_4_)/graphene nanoribbon/graphene (LFP/GNR/G) paper-like binder-free electrode [42]. Spray deposition technology ensures uniform component distribution in each droplet of the precursor slurry, which leads to good composite membrane uniformity.

The electrostatic-based technique is a typical low-cost and highly effective assembly method. Electrodeposition (ED) can be used as a simple and effective method to prepare ultrathin and highly ordered nanosheets. For instance, using an electrodeposition process, a three-dimensional (3D) porous self-supporting molybdenum sulfide/graphene (MoS_x_/G) composite film was fabricated [43], in which the graphene nanosheets were anchored on nanogranuled MoS_x_ particles. This film showed a well-developed porous structure and exceptional electrical performance. Electrophoretic deposition (EPD), another typical material-processing technique, entails a two-step process: the charged particles in a suspension are driven toward an electrode of opposite charge due to the influence of an electric field and are then deposited to form a compact film. Yang et al. developed a cobaltosic oxide/graphene (Co_3_O_4_/G) hybrid electrode by electrophoretic deposition [44]. Due to the excellent flexibility of graphene and the large number of voids in this sandwich-like structure, the structural integrity and unobstructed conductive network can be maintained during cycling. Electrospinning is another kind of widely used fiber production method that relies on electric force guidance. As a typical example, Niu et al. designed a gradient electrospinning method to produce mesoporous nanotubes and pea-like nanotubes that showed higher ionic and electronic conductivities and larger specific surface areas compared to traditional nanowires [45].

Magnetic field is a strong force capable of aligning ferromagnetic materials [46]. Magnetic-field-induced self-assembly is an effective strategy for the construction of micro–nano-ordered structures [47–49]. By controlling the magnetic field direction and the magnetic field line distribution, various ordered structural materials can be obtained simply and quickly [50, 51]. For example, a magnetite reduced graphene oxide (rGO)/Fe_3_O_4_ hybrid paper has been fabricated at the air/liquid interface, assisted by an external magnetic field, which achieved uniform size distribution and monodispersibility of Fe_3_O_4_ nanoparticles and avoided the agglomeration of graphene oxide sheets [52]. Additionally, through electroless deposition with a magnetic field and further annealing, Kawamori et al. prepared a nickel oxide (NiO)-covered nickel nanowire nonwoven cloth, which showed quite high cyclability as an electrode material, without binders, conductive additives, or current collectors [53].

Microwaves, another source of external force, also have a great effect on the carbon assembly processes. Normally, CVD methods can be used to synthesize conductive matrixes [54] or deposite other active materials [55] to form hybrid nanostructures. In particular, with the assistance of microwaves, vertically aligned structures can also be fabricated through CVD processes. For example, using a microwave plasma-enhanced CVD system and hydrogen fluoride (HF)-CVD technology, Gohier et al. synthesized vertically aligned CNTs decorated with Si-particle arrays (VA-CNTs/Si) [56]. This well-designed structure not only ensured the dispersion uniformity of the Si nanoparticles, but also provided short 3D transportation pathways for both lithium ions and electrons. Toward the optimization of material structures and organizations during preparation, the introduction of mechanical forces, such as in the typical high-energy ball-milling approach, is another common low-cost method. Huang et al. produced nanostructured phosphorus (P)-doped Si/graphite composites using a two-step ball-milling method, which exhibited fast transport for both lithium ions and electrons [57]. Ball milling is a cost-effective and easily scalable method, which makes it feasible for large-scale carbon-based material synthesis applications.

It is noted that, during the fabrication of carbon–noncarbon composites, additional complicated preparation processes for the carbon architectures are generally needed. Efforts have focused on one-step methods of preparing carbon–noncarbon hybrid architectures that not only simplify the preparation process to save time, but also result in high pack densities and closer contacts between the carbon and noncarbon materials [58]. In the one-step construction of carbon–noncarbon hybrid architectures, the assembly of the carbon buildings and the production of noncarbon components occur simultaneously; hence good mixing of the carbon and noncarbon precursors, and the subsequent co-production of the carbon and noncarbon components, are necessary in the overall fabrication process.

## Enhanced Roles of Carbon Architectures in LIBs

In a lithium-ion battery, the electrode side typically consists of an active material (anodic or cathodic material), a conductive carbon matrix, a binder, and a current collector, each of which impacts significantly on the whole battery performance. Carbon is a critical component of current LIBs, especially as active materials for lithium storage or conductive matrixes that accelerate electron transfer. Nevertheless, much effort needs to be dedicated to the assembly of the active carbon material to improve charge transport across thick electrodes. Furthermore, with the challenge of promoting practical applications of high-capacity noncarbon anode materials, the carbon should be well engineered to solve the emerging issues of structural and interfacial instabilities and side reactions with the electrolyte. In this part, a variety of advanced assembly strategies that reinforce the roles of the carbon architectures as active materials, electrochemical reaction frameworks, and current collectors in electrodes for high-performance and long-cycling LIBs, are fully discussed.

### High-Rate-Performance Carbonaceous Active Materials

**Fig. 3:**
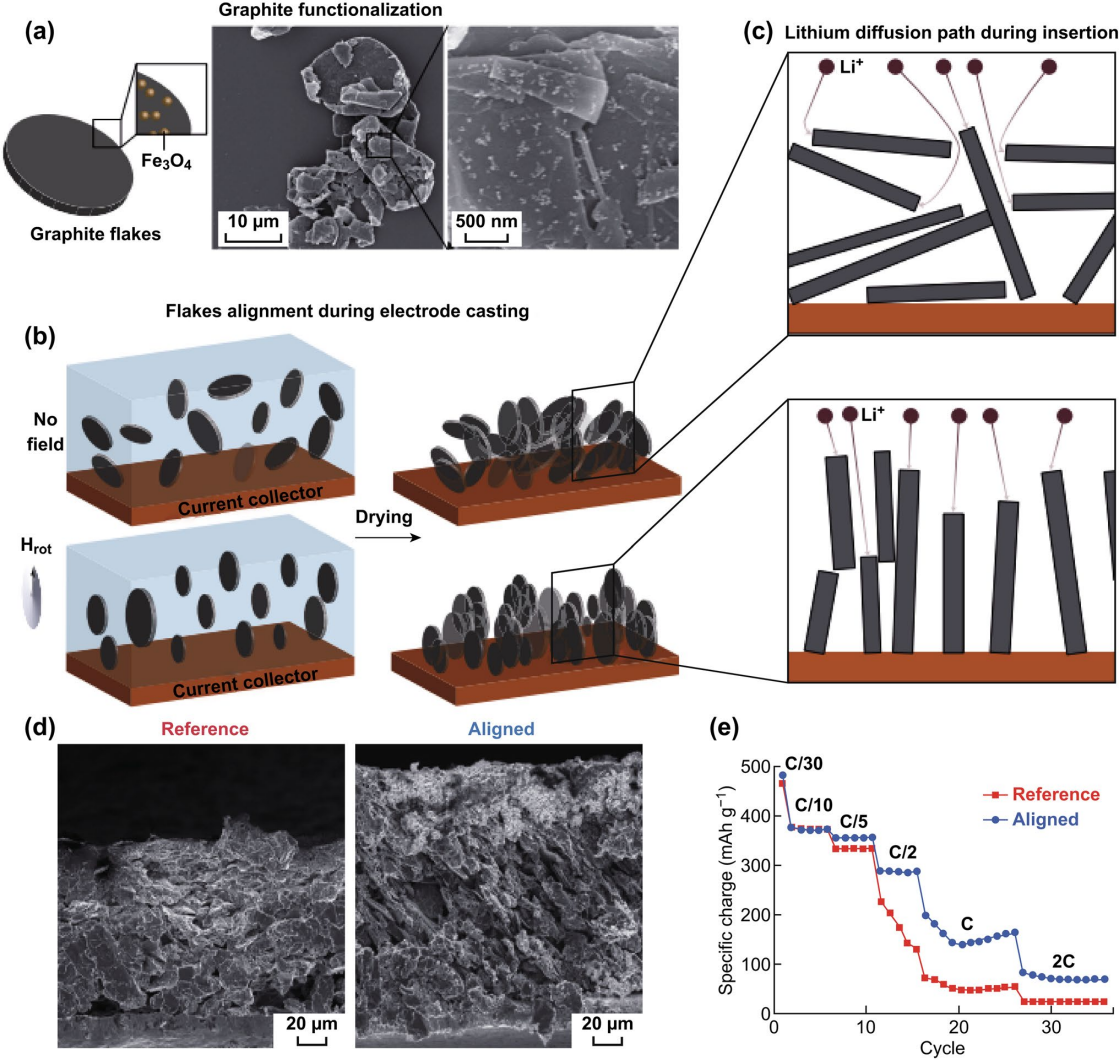
**a** Sketch and SEM images of graphite flakes coated with Fe_3_O_4_ nanoparticles. **b** Graphite-electrode casting. **c** Sketch depicting lithium diffusion pathways in both electrode architectures. **d** Rate capability. Reprinted with permission from Ref. [62]

Carbon materials have been widely investigated as lithium-ion battery anode materials due to numerous merits, including their excellent electrical conductivities, superb chemical stabilities, high surface areas, light weights, and tunable porous structures. Significant research has focused on advanced and well-designed carbon-based materials, from the microscale to the nanoscale [59–61]. As the currently used anode material in commercial LIBs, graphite possesses excellent structural stability and reaction reversibility during lithium-ion intercalation/deintercalation processes. Nevertheless, further improving the fast-charging capabilities of thick graphite anodes remains challenging. The directional assembly of graphite flakes to reduce tortuosity is a very effective way of improving the diffusion kinetics of lithium ions in graphite anodes. With the assistance of a magnetic field, Juliette et al. orientationally controlled anodic graphite flakes to achieve high mass loadings and excellent rate performance in LIBs (Fig. 3a–c) [62]. Coated with superparamagnetic nanoparticles, graphite flakes were engineered into a thick (~ 200 μm) and highly loaded (~ 10 mg cm^−2^) electrode with an out-of-plane-aligned architecture under a rotating magnetic field. As a result, this highly load yet poorly tortuous graphite anode delivered a specific capacity (~ 200 mAh g^−1^) that was three times higher than that of a conventional anode at a high charge/discharge rate of 1 C (Fig. 3d). Promisingly, magnetic field guidance during the assembly of electrode materials is a rapid method that is scalable to practical electrode engineering, and can form the basis for new fabrication processes that enable the fabrication of thick, inexpensive, and high-power-density electrodes.

The assembly of carbon building blocks into 3D carbon networks also plays a significant role in enhancing the rate performance of carbon anodes, especially for graphene-derived structures [63]. Wang et al. fabricated a high density (1.1 g cm^−3^) N-doped holey-graphene monolith (NHGM) using a one-pot hydrothermal process with hydrogen peroxide (H_2_O_2_) as an added etching agent [64]. Due to efficient diffusion channels for lithium ions across graphene planes, highly conductive pathways for electrons, and incremental edges on sheets that enhanced lithium-ion intercalation, the as-prepared NHGM delivered a high volumetric capacity above 800 mAh cm^−3^ at a high rate of 3.2 mA cm^−2^, and with a high mass loading of 2.75 mg cm^−2^. Inspired by branched structures in nature, a highly branched 3D N-doped graphitic (BNG) tubular foam was fabricated using template-assisted single-source *N, N*-dimethylformamide (DMF)-CVD technology [65]. Homogeneous N-doping in the branched 3D-BNG not only produced defects in both the flat and curved parts that contributed to increased capacity, but also enlarged the spacing distances in both the flat and curved parts (≈ 0.40 nm), leading to a better rate capability. Consequently, when acting as the anode material in a lithium-ion battery, the BNG foam showed remarkably superior capacity (1049 mAh g^−1^ at 500 mA g^−1^), excellent cycling performance (725 mAh g^−1^ even after 200 cycles at 1500 mA g^−1^), and enhanced rate performance (451 mAh g^−1^ at 2.09 C).

### High-Efficiency Carbon-Based Electrochemical Reaction Frameworks

To further increase the energy densities of LIBs, many researchers have focused on high-capacity anode materials, including alloy-based anode materials (e.g., Si, Sn, and Ge) [66–68], metal oxides (e.g., Co_3_O_4_, Fe_3_O_4_, and SnO_2_) [69–72], and layered metal dichalcogenides (MX_2_, e.g., MoS_2_ and WS_2_) [73, 74], to replace low-capacity graphitic anode materials. However, these active electrode materials with high specific capacities have low intrinsic electronic conductivities and suffer from drastic lithiation-induced volume expansions, which prevent their use in practical LIBs [75].

Carbons, used as second phases, are generally introduced into noncarbon materials to achieve promisingly high capacities and long-term cycling performance [76–78]. Furthermore, architectures constructed using carbon building blocks not only offer large surface areas to load noncarbon active particles, but also provide interconnected conductive networks, effective protective layers, and sufficient void spaces for noncarbons during cycling. As the supporting electrochemical matrix, the structural design of the carbon component significantly influences the electrochemical performance of the electrode. Thus, in the following section, we discuss the roles of carbon architectures in terms of interfacial optimization, network construction, and void space and densification designs, which are equally important for improving electronic and ionic conductivities, Coulombic efficiencies, packing densities, and the structural stabilities of high-capacity noncarbon electrodes.

#### Interfacial Modulation

For the construction of carbon–noncarbon hybrid electrode materials for LIBs, the interfacial design and the carbon–noncarbon interaction model following assembly of the active materials into various kinds of carbon matrix (such as CNTs [79], graphene sheets [80], amorphous carbon layers [81], or graphitic carbon [82]), greatly influence charge transport and the structural stability of the hybrid. For a battery, fast electron transport is a vital factor for high electrical performance. Normally, to improve the conductivities of noncarbon materials, carbon supports or coatings are introduced, after which carbon–noncarbon hybrids with inner fast electron highways are formed. As a typical example, the conventional conductive carbon additives (carbon black and conducting graphite) used to improve electron transport in electrodes rely on the formation of model “point-to-point” contacts, whereas our group developed a “plane-to-point” contact model using 2D graphene as the conductive additive, which greatly promotes the electrochemical performance of low-conductivity noncarbon electrodes [83]. Furthermore, to avoid steric effects during ion diffusion in 2D graphene, especially for thick electrodes, a combination of “point-to-point” and “plane-to-plane” contact models can be used to form a synergistic conductive network for noncarbon materials [84].

**Fig. 4:**
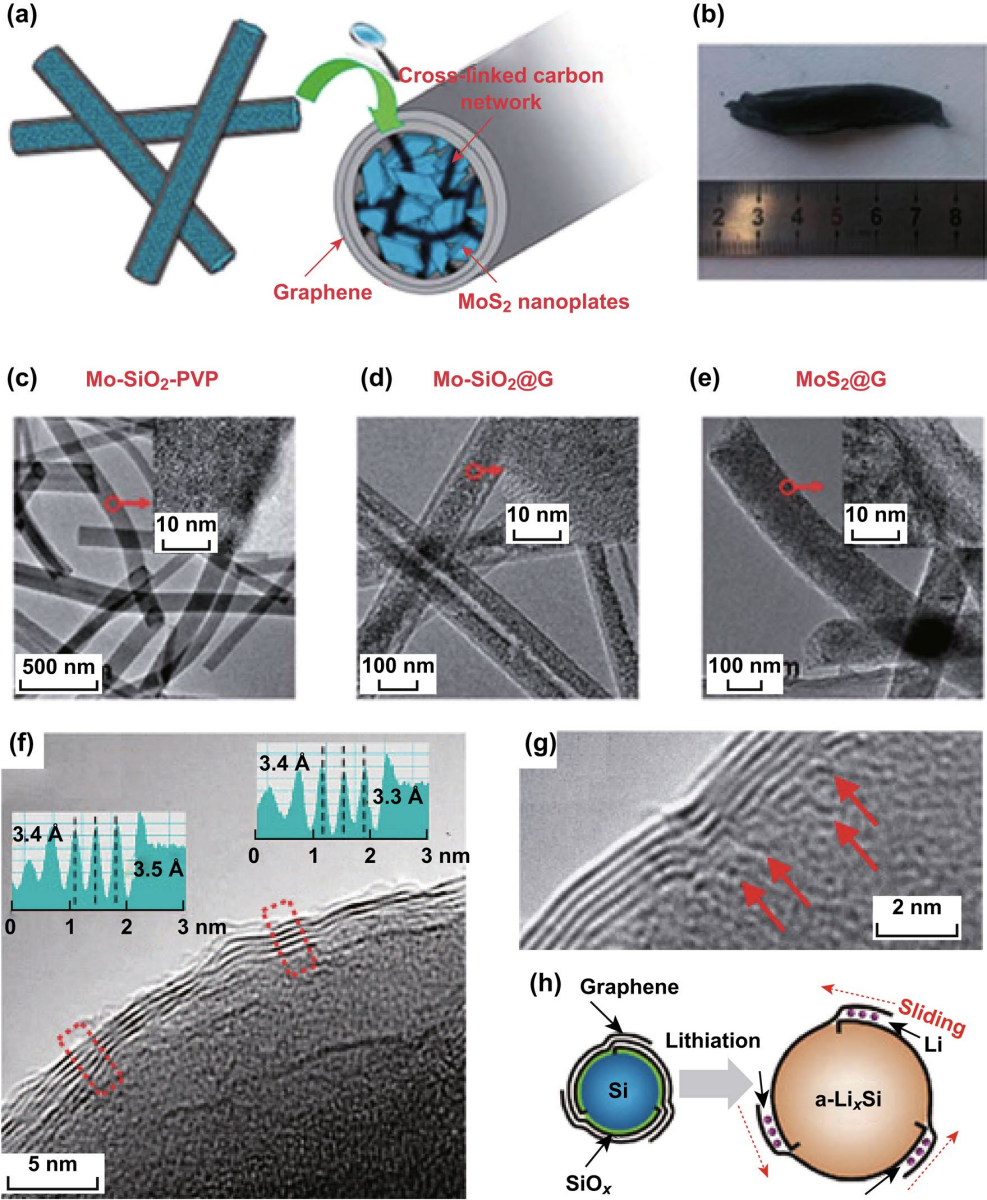
**a** Schematic of MoS_2_@G. **b** Free-standing MoS_2_@G membrane. SEM images of **c** Mo-SiO_2_-PVP, **d** Mo-SiO_2_@G, and **e** MoS_2_@G. Reprinted with permission from Ref. [87]. **f** HRTEM image of Gr-Si NP. **g** HRTEM image visualizing the origins (red arrows) of individual graphene-layer growth. **h** Schematic illustration showing the sliding of graphene coating layers that buffer the volume expansion of Si. Reprinted with permission from Ref. [88]

Apart from the above-mentioned two contact conductive models involving carbons and noncarbons, face-to-face interactions also play critical roles in enhancing the electrochemical performance of noncarbon anodes. Typically, face-to-face interactions between 2D Si nanosheets and thin carbon layers provided excellent electronic/ionic pathways as well as good structural stabilities, leading to improved lithium-ion storage performance [85]. For instance, layers of Si were deposited onto 3D graphene/CNT aerogels (CAs) to form Si/CA nanohybrids through simple CVD processes [86]. In these Si/CA nanohybrids, face-to-face contacts between Si and graphene led to rapid electronic/ionic transfer and create sufficient voids, which contributed to high structural integrity. Therefore, compared with a Si/graphene–CNT mixture, this Si/CA nanohybrid delivered much higher reversible capacity (1498 mAh g^−1^ at 200 mA g^−1^) and a more remarkable rate capability (462 mAh g^−1^ at 10 A g^−1^). Moreover, to increase the mass content of the active materials and the stabilities of high-capacity anodes, Kong et al. developed a novel “side-to-face” contact model, in which electrochemically active molybdenum disulfide (MoS_2_) nanosheets directly stood on the inner surfaces of graphitic nanotubes, thereby forming mechanically robust, free standing, and interwoven MoS_2_@G nanocable webs (Fig. 4a–e) [87]. The effective interfacial contact between the MoS_2_ nanosheets and the graphitic nanotubes not only realized full use of the active substance (MoS_2_), but also facilitated the use of less carbon without the degradation of electrical performance, even at ultrahigh MoS_2_ content (90%). Remarkably, a high specific capacity of 1150 mAh g^−1^, an excellent cycling capability (~ 100% capacity retention after 160 cycles), and an ultrahigh rate performance of 700 mAh g^−1^ at a current density of 10 A g^−1^ were obtained using this MoS_2_@G hybrid material with such a high MoS_2_ loading. To buffer dramatic volume expansion and further improve the volumetric performance of high-capacity noncarbons, Son et al. reported dynamic interfacial interactions between graphene and Si nanoparticles (Fig. 4f–h) [88]. In this work, a structure in which graphene layers were anchored onto a Si surface (Gr–Si NPs) was obtained by employing the CVD approach, which introduced a novel graphene-interlayer sliding process that buffered Si expansion upon lithiation and delithiation. With this dynamic encapsulation, the well-defined conductive graphene coating enabled fast electron transport, while the layered graphene structure also relieved volume expansion to a large extent. Consequently, it exhibited a high volumetric energy density of 700 Wh L^−1^ even after 200 cycles, which was 1.5 times higher than that of a graphite-based control cell (471 Wh L^−1^).

#### Network Skeleton Design

Besides interfacial interactions as factors in carbon architecture design, the formation of a continuous carbon network is also of critical significance for facilitating ion and electron transport, and for buffering volume expansion involving noncarbon particles. In particular, to solve the challenges of poor charge transport and huge volume fluctuation in dense and thick electrodes, a variety of carbon networks with directional, graded, and hierarchical features have been rationally designed for high-capacity noncarbon electrodes in recent research.

As discussed above for the directional assembly of active carbon materials, the directional transport of ions can also be achieved in carbon–noncarbon electrodes by vertically constructing a carbon network. Several pieces of work have been devoted to the design and synthesis of vertically aligned graphene arrays (VAGAs) [19] or graphene-based arrays, such as Si NPS@graphene nanosheets [89], germanium oxide (GeO_x_) on VAGAs [90], tin@graphene (Sn@G) on VAGAs [91], and MoS_2_ on vertical graphene nanosheets (VGNS) [92], for use in LIBs. Typically, the vertically aligned MoS_2_/VGNS nanostructure [92], fabricated through CVD and solvothermal processes, showed a high specific capacity (1277 mAh g^−1^ at 100 mA g^−1^) and excellent cycling stability (1109 mAh g^−1^ after 100 cycles). This excellent electrochemical performance mainly originated from the directional carbon-based architecture, which not only provided fast electron and lithium-ion diffusion pathways, but also achieved stable structural integrity during cycling.

Compared to the design of homogeneous electrodes, producing a functionally layer-graded electrode is another effective strategy for promoting charge-carrier transport. Zhang et al. demonstrated a functionally layer-graded electrode composed of titanium dioxide (B) and multilayered rGO nanotubes that reduced the charge-transport barrier [93]. Consequently, this layer-graded electrode showed a much higher rate performance (128 mAh g^−1^ at 20 C) compared with a traditional homogeneous electrode (74 mAh g^−1^ at 20 C) on account of the synergism between the reduction in the lithium-ion diffusion energy barrier and the improvement in electronic conductivity.

Multiple hierarchical core/shell directional arrays composed of two or more components, in which 2D building blocks were vertically grown on 2D/3D conductive substrates that can serve as self-supported electrodes, were fabricated to ensure charge delivery across the whole electrode. Firstly, direct contact between the active components and the surfaces of conductive substrates guarantees rapid electron transfer. Additionally, the gap between each array accommodates large volume expansion and contraction upon lithiation and delithiation. Using a simple surfactant-assisted hydrothermal method combined with post-annealing treatment, Shen et al. synthesized a structure composed of mesoporous nickel cobaltate (NiCo_2_O_4_) nanowire arrays (NWAs) anchored on carbon textiles [94]. When directly used as a binder-free electrode in a lithium-ion battery, this flexible NiCo_2_O_4_/carbon composite textile exhibited high capacity (1012 mAh g^−1^ at 0.5 A g^−1^), good cycling stability (retaining 854 mAh g^−1^ at 0.5 A g^−1^ after 100 cycles), and excellent rate performance (778 mAh g^−1^ at 2 A g^−1^). The excellent performance of this composite textile was mainly ascribable to the ample mesoporous structure of the nanowire array and the large open spaces between neighboring nanowires, which ensured that all nanowires participate in ultrafast electrochemical reactions and alleviated volume changes during the charge/discharge processes. Wang et al. presented a strategy that involves patterning vertical MoS_2_ nanosheets onto electrochemically exfoliated graphene (EG) to achieve a hierarchical architecture [95]. Using synergism between the vertically aligned structure and the 2D geometry, this structure exhibited high mechanical integrity and fast charge-transport kinetics. When serving as an anode material in a lithium-ion battery, it delivered an ultrahigh specific capacity of 1250 mAh g^−1^ after 150 stable cycles at 1 A g^−1^, excellent rate performance of 970 mAh g^−1^ at 5 A g^−1^, and a high areal capacity of 1.27 mAh cm^−2^ at ~ 1 mA cm^−2^.

**Fig. 5:**
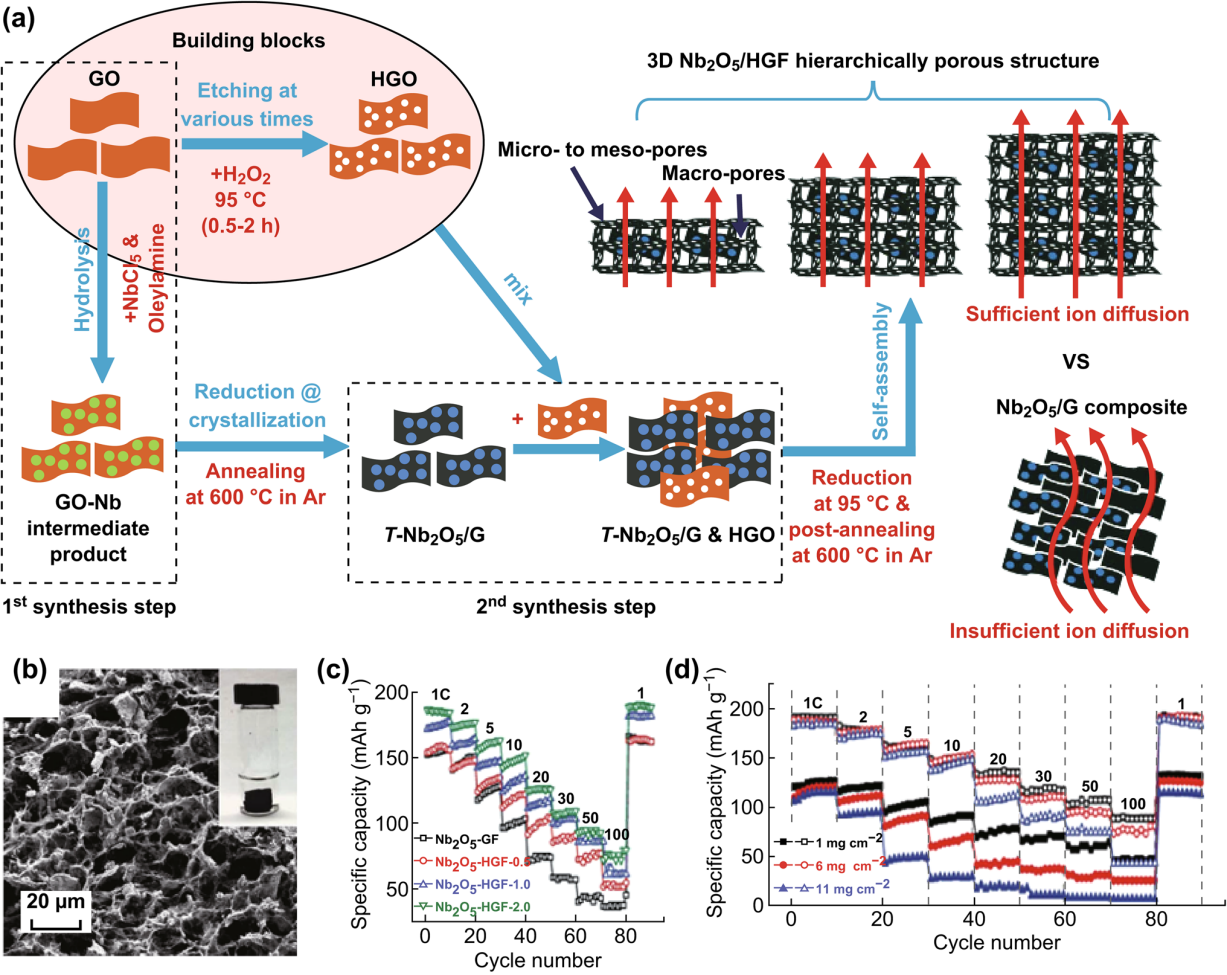
**a** Schematic of the fabrication process. **b** Cross-sectional SEM image. (Inset) A free-standing monolithic electrode. **c** Rate capability at tunable nanoporosity. **d** Rate performance at different mass loadings. Reprinted with permission from Ref. [97]

The assembly of holey or porous carbon building blocks into hierarchical networks plays a significant role in enhancing ion-transport kinetics for thick electrodes upon cycling. For example, Wang et al. synthesized a 3D carbon nanosheet array/MnO hybrid (3D-MnO/CNS). MnO nanocrystallites were mechanically anchored in this unique structure by the pore-surface terminations of the 3D arrays of graphene-like carbon nanosheets, resulting in both fast electron transport and a 20-nm-scale diffusion distance in this interlinked architecture. As a result, a high lithium storage capacity (1332 mAh g^−1^ at 0.1 A g^−1^) and a high rate performance (285 mAh g^−1^ at 20 A g^−1^) were delivered, with stable cycling performance (500 cycles) [96]. To promote the use of nanostructured electrodes in high-energy and high-power LIBs, Sun et al. reported the design of a 3D holey-graphene/niobia (Nb_2_O_5_) composite for ultrahigh rate energy storage at practical levels of mass loading (> 10 mg m^−2^) [97]. As shown in Fig. 5a, the two-step process for the preparation of the free-standing Nb_2_O_5_/HGF composite included the preparation of building blocks and the assembly of these blocks into the 3D holey monolithic composite (Fig. 5b). The pore size of the GO plane can be easily tuned by changing the H_2_O_2_-etching time, thereby providing suitable ion pathways. In this hybrid material, the 3D graphene network provided an interconnected conductive network, and its hierarchical porous structure facilitated rapid ion transport and mitigated diffusion limitations throughout the entire electrode architecture. There was little difference in specific capacity for mass loadings ranging from 1 to 11 mg cm^−2^ at rates as high as 10 C (Fig. 5c–d). Promisingly, the Nb_2_O_5_/HGF composite electrode showed high areal capacity with high rate capability at large mass loadings, which represented a critical step toward the practical application of high-power-density LIBs.

The construction of hierarchical carbon networks can greatly alleviate volume variations of the noncarbon components and help to avoid side reactions with the electrolyte. Liu et al. presented a classic “pomegranate”-like hierarchical Si structure, which effectively tackled the major problems of structural failure, the repetitive formation of solid electrolyte interface (SEI) layers, and the low volumetric capacities of Si anodes [30]. Recently, Xu et al. designed and synthesized hierarchical Si/C hybrids inspired by the structure of watermelon fruit [98]. Due to the dual protection strategy of a hierarchically structured buffer and an optimized particle-size distribution, this Si/C anode afforded excellent electrochemical performance, including a superior average coulombic efficiency of 99.8% during early cycles, and a good cyclic life of 500 cycles under practical mass loadings and pressing densities.

#### Void Space Manipulation

To address the problem of volume expansion, the design of the void space in a carbon–noncarbon hybrid structure is critically important. Tremendous work has focused on fabricating hollow structures, such as C@manganese oxide (MnO_2_) yolk-shelled spheres [35] and multi-shelled nickel sulfide (NiS) nanoboxes [99]. Typically, Fe_3_O_4_@iron carbide (Fe_3_C)@C yolk-shelled nanospindles were synthesized using a one-step in situ nanospace-confined pyrolysis strategy involving Fe_2_O_3_@resorcinol–formaldehyde (RF) core@shell nanospindles [100]. This unique structure provided sufficient internal void space for electrochemically active Fe_3_O_4_ and afforded a Fe_3_C/C dual shell that restricts Fe_3_O_4_ dissolution. Consequently, this nanospindle exhibited a high reversible capacity of 1128 mAh g^−1^ in a lithium-ion battery. Compared to typical yolk spheres, the yolks in polyhedral shells had larger contact areas that facilitated conducting electrons and diffusing ions. Zhang et al. synthesized a yolk Sn@C nanobox composite with controllable shell thickness by a novel method [101]; zinc stannate (ZnSnO_3_) nanocubes as precursors were uniformly coated with polydopamine (PDA), after which they thermally treated to carbonize the PDA and reduce ZnSnO_3_ to metallic Zn/Sn in situ. After evaporation of the low-boiling Zn, a yolk-shell Sn@C nanobox was finally produced. The void space was easily tuned to fit the volume expansion of Sn during lithiation and delithiation by controlling the concentration of PDA. Thus, with a suitable shell thickness, the resulting composite exhibited a high reversible capacity of 810 mAh g^−1^, even after 500 cycles, and benefited from a sufficient buffer for volume change during cycling. To solve the structural fracturing of micro-Si particles, Li et al. fabricated a unique Si/C hybrid microparticle in which mechanically strong and flexible graphene cages completely encapsulate micrometer-sized Si particles [102]. During preparation, a dual-purpose Ni template acted as both the catalyst for graphene growth and the sacrificial layer that provided void space. The graphene cage not only possessed excellent electrical conductivity and adequate buffer space, but also promised electrical connectivity for fractured Si microparticles after repeated lithiation. In addition, efficient SEI formation on the surface of the graphene cage minimized irreversible lithium ion losses, leading to high coulombic efficiencies (93.2% and 99.9% respectively) during initial and subsequent cycles. When paired with a traditional lithium cobalt oxide (LCO) cathode, the graphene-caged Si microparticles showed excellent stable cycling (100 cycles; 90% capacity retention) during full-cell electrochemical testing.

Except for the synthesis of carbon cages or shells that surround single electroactive particles, the fabrication of 3D interconnected porous carbon structures is another common strategy for introducing void space. Jung et al. synthesized a Si-carbon composite (Si@po-C) using an industrially established spray-drying process, in which Si nanoparticles were uniformly distributed in porous carbon spheres [103]. The porous structures inside the carbon spheres provided void space for the volume expansion of Si, as well as improved electrical and ionic conductivity, which was conducive to excellent electrochemical performance, including 1956 mAh g^−1^ at a 0.05 C rate and a 91% capacity retention after 150 cycles.

**Fig. 6:**
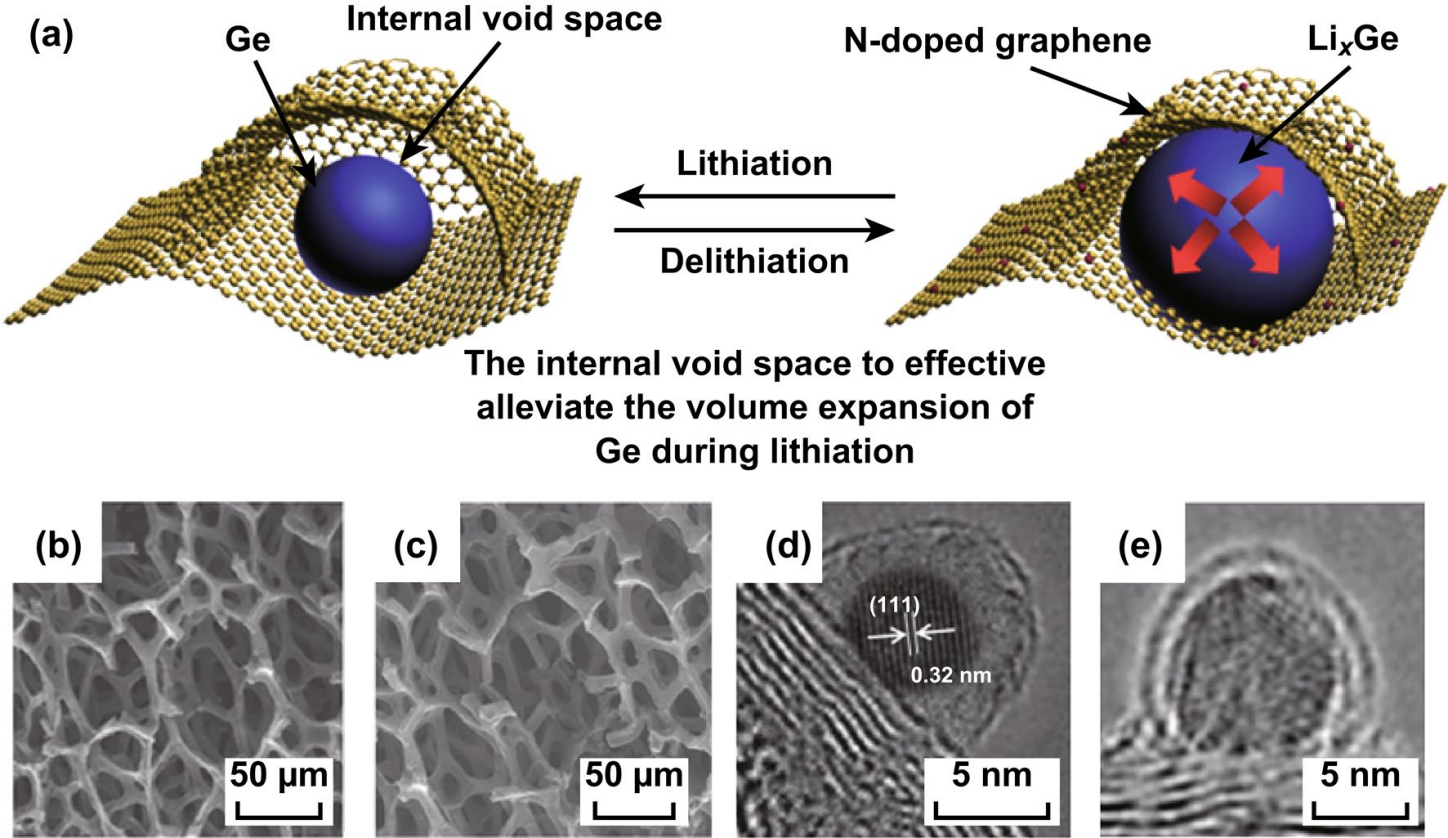
**a** Schematic of the lithiation/delithiation processes of the Ge-QD@NG/NGF/PDMS yolk-shell electrode. **b**, **c** SEM and **d**, **e** TEM images before cycling and under lithiation after 1000 cycles at a current density of 1 C. Reprinted with permission from Ref. [104]

Several pieces of advanced work have focused on 3D free-standing structures that can be directly used as binder-free electrodes. For example, Mo et al. developed a 3D interconnected porous nitrogen-doped graphene foam (NGF) with an encapsulated Ge quantum-dot@nitrogen-doped graphene yolk-shell nanoarchitecture [104]. This unique 3D nanoarchitecture not only afforded internal void space for accommodating the huge volume change of Ge during cycling, but also provided ample channels for access to the electrolyte, an interconnected conductive network, and fast lithium-ion diffusion pathways (Fig. 6). Hence, this 3D yolk-shell nanoarchitecture exhibited excellent electrical performance, including a high specific reversible capacity of 1220 mAh g^−1^, long-cycling capability (over 96% capacity retention from the second to the 1000th cycle), and an ultrahigh rate performance (over 800 mAh g^−1^ at 40 C). Ma et al. encapsulated Si nanoparticles into highly oriented graphene foam (GF) using a freeze-drying method [105]. Rich inner pores in the GF promised well-accessible channels for electrolyte penetration and electron/ion transportation; they also tackled the issues of huge volume changes and mechanical instability during charge/discharge processes. Consequently, this GF/Si composite displayed stable cycling performance in both half-cell (retaining 1170 mAh g^−1^ at 1 A after 1200 cycles) and full-cell (90% capacity retention over 200 cycles) configurations.

#### Densification Engineering

The nanostructured carbons used for electrode construction can significantly improve mass-based capacity and rate performance, but unfortunately, they usually lead to low volume-based capacities due to the low densities of carbon-based active materials [15]. Meanwhile, the abundant void spaces introduced into the carbon architectures to buffer the volume expansion of noncarbons makes it difficult to attain high volumetric performance. As a result, those nanostructures with high mass capacities but low packing densities deliver low specific volumetric energy densities, which limit their practical applications. The main remedy for this issue involves increasing the particle size of the electroactive material to avoid low tap density [106]. Much research has focused on building secondary structures composed of agglomerated nanosized primary particles based on carbon assemblies, which are capable of not only achieving high electrode density, but also preserving the electrochemical properties of the nanosized active materials [107].

**Fig. 7:**
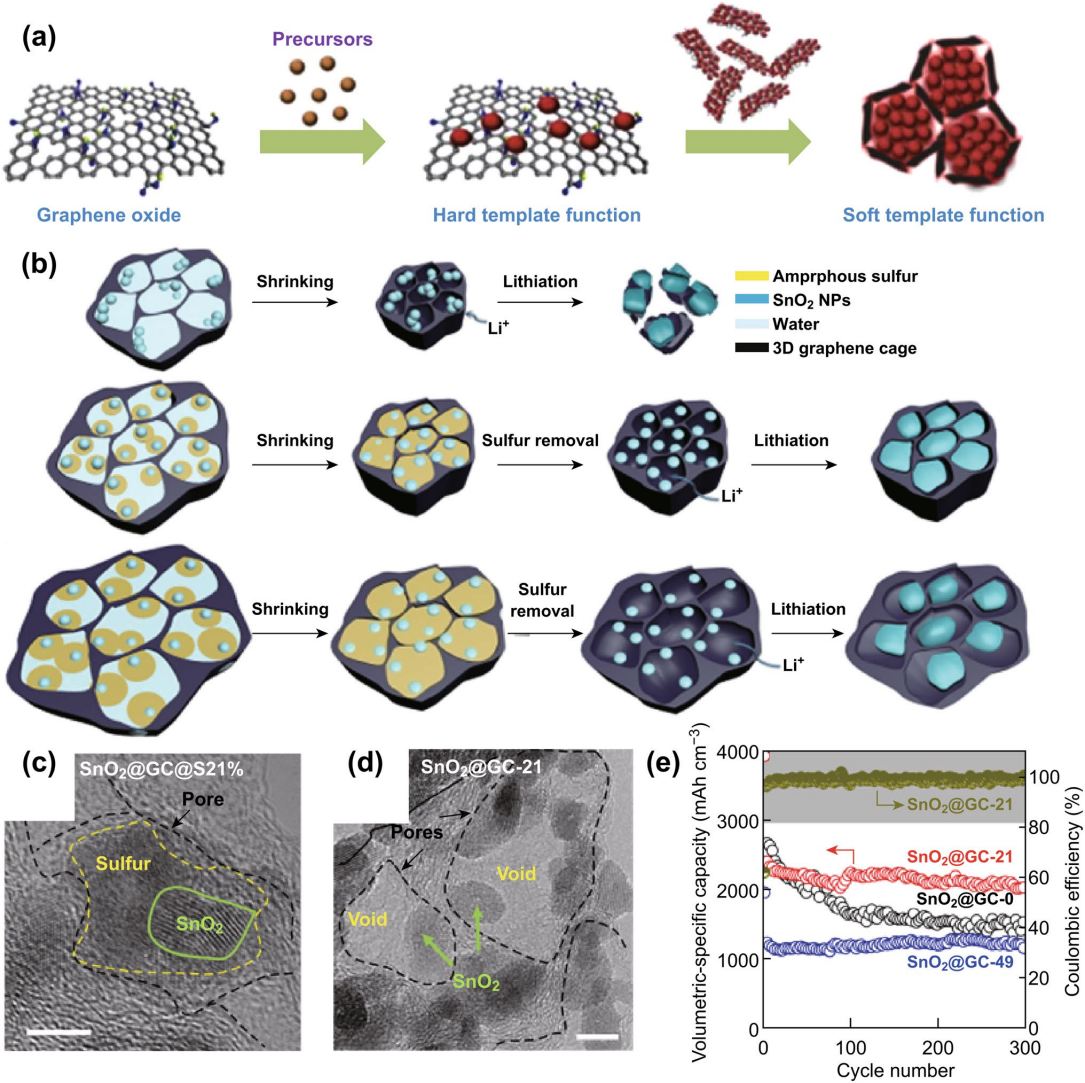
**a** Schematic of in situ nanocrystal growth with GO as both hard and soft templates. Reprinted with permission from Ref. [108]. **b** Schematic of the sulfur-template control strategy that incorporates void space. HRTEM image of SnO_2_@GC: **c** before sulfur removal and **d** after removal of all of the original sulfur (Scale bar: 5 nm). **e** Cycling performance. Reprinted with permission from Ref. [113]

For example, by using graphene oxide (GO) as both hard and soft templates [22], our group fabricated a high packing density (1.35 g cm^−3^) ferric oxide/graphene microparticle hybrid (Fe_2_O_3_-G) [108]. Two-dimensional GO sheets provided abundant nucleation sites for Fe_2_O_3_ growth and the formation of Fe_2_O_3_ microspheres, and hydrothermal treatment afforded a 3D interconnected graphene conductive framework for embedded Fe_2_O_3_ microspheres (Fig. 7a). This Fe_2_O_3_-G microsphere design not only provided an efficient conductive network and sufficient void space to alleviate large Fe_2_O_3_ volume changes, but it also ensured high material tap densities. As a result, these Fe_2_O_3_-G microspheres concurrently delivered a high capacity, excellent rate performance, and a super-high volumetric capacity of about 1200 mAh cm^−3^ (three times that of a commercial graphite anode (370 mAh cm^−3^)).

Nanosized spinel cathode (LMO) materials possess good intrinsic rate capabilities but cannot fulfill the requirements of high electrode densities and volumetric energy densities. Carbon coating has been widely used to increase electronic conductivity, but traditional carbon coating by the pyrolysis of organic compounds or CVD on the LMO surface unavoidably always produces oxygen defects, which leads to a blocked rate capability. To solve this problem, Lee et al. fabricated spherical secondary particles composed of acid-treated Super P (ASP) and nanosized LMO primary particles using a water-based spray-drying process, referred to as “ASPLMO” [16]. In this composited material, the nanosized LMO primary particles ensured fast lithium-ion diffusion and uniformly dispersed conductive pathways, while the microsized structure (7–40 μm) guaranteed a high electrode density (2.4 g cm^−3^). Consequently, this ASPLMO material exhibited a volumetric energy density of 270 Wh L^−1^ at a power density of 780 kW L^−1^. Through a one-pot mixed-solvothermal process followed by direct calcination, Wang et al. synthesized LiFePO_4_@carbon/rGO hierarchical microspheres [109] that exhibited a high tap density of 1.3 g cm^−3^, while the carbon layers coated on LiFePO_4_ primary nanocrystals and ultrathin rGO nanosheets anchored on the surface simultaneously provided fast electron/lithium-ion transport, resulting in excellent rate capability and cycling stability.

Apart from the increase in the particle size of the active material, the densification of carbon network and the hybridization of high-density and high-capacity noncarbons are further simple but effective strategies for improving the packing densities of carbon-based electrode materials [110, 111]. The compact assembly of graphene, free of inter-particle voids, has shown great potential for high volumetric energy storage [112]. As a typical example, a hybrid hydrogel of a noncarbon (Sn and Si-based particles) and a 3D graphene network was synthesized by a hydrothermal process; this hydrogel was subsequently treated by capillary drying to achieve shrinkage, to yield a 3D ultrahigh-density assembly. This method can easily be extended to the densification of graphene-based materials derived from hydrogels; however, it is difficult to ensure that sufficient void space is left for noncarbon-particle expansion upon lithiation. Recently, our group chose sulfur as a flowable template for the preparation of a tin oxide@graphene cage hybrid (SnO_2_@GC) with a high packing density, yet well-defined void space (Fig. 7b–d) [113]. Smart sulfur can seamlessly encapsulate noncarbon particles following the capillary drying of graphene hydrogels. The content of this sacrificial sulfur agent can be used to precisely adjust the quantity and sizes of the pores, resulting in different sizes of carbon cages following sulfur removal. When used as an anode material in a lithium-ion battery, the resultant graphene-caged SnO_2_ afforded a high specific capacity of 974 mAh g^−1^ and an ultrahigh volumetric capacity of 2123 mAh cm^−3^, together with a long cycle life (300 cycles) and limited change in electrode thickness (< 20%) (Fig. 7e). Our result suggests that this general strategy of precisely tailoring the buffer space in a carbon cage not only provides sufficient voids for volume change, but also engineers the packing density to produce high energy storage capabilities in small volumes.

### Highly Contacted Carbon-Based Current Collectors

Besides focusing on accelerating charge-carrier transport and enhancing structural stability at the electrochemically active material level by the carbon assembly strategy, the design of carbon architectures as current collectors also contributes to improving electrochemical performance. Well-assembled lightweight carbon architectures can afford stronger interfacial interactions, larger contact areas, and greater charge delivery at both the active material and electrode level than conventional copper (Cu) and aluminum (Al) current collectors, which is highly desirable for improving specific capacity, rate performance, and structural stabilities of high-capacity noncarbon electrodes.

A few strategies have been developed to maintain strong electrical connections and achieve fast electron/ion transport inside an electrode, by engineering carbon materials into current collector matrixes [114]. Rather than traditional Cu or Al current collectors, carbon-based current collectors are lighter and more flexible. For example, Wu et al. fabricated a CNT woven macrofilm (CMF) that acted as current collector through methanol-mediated CVD [115]. The active materials were permeated into the surface of the CMF, thereby alleviating delamination under ultrahigh mass loadings (10 mg cm^−2^) and contributing to a high-energy density of 215 mWh cm^−3^.

**Fig. 8:**
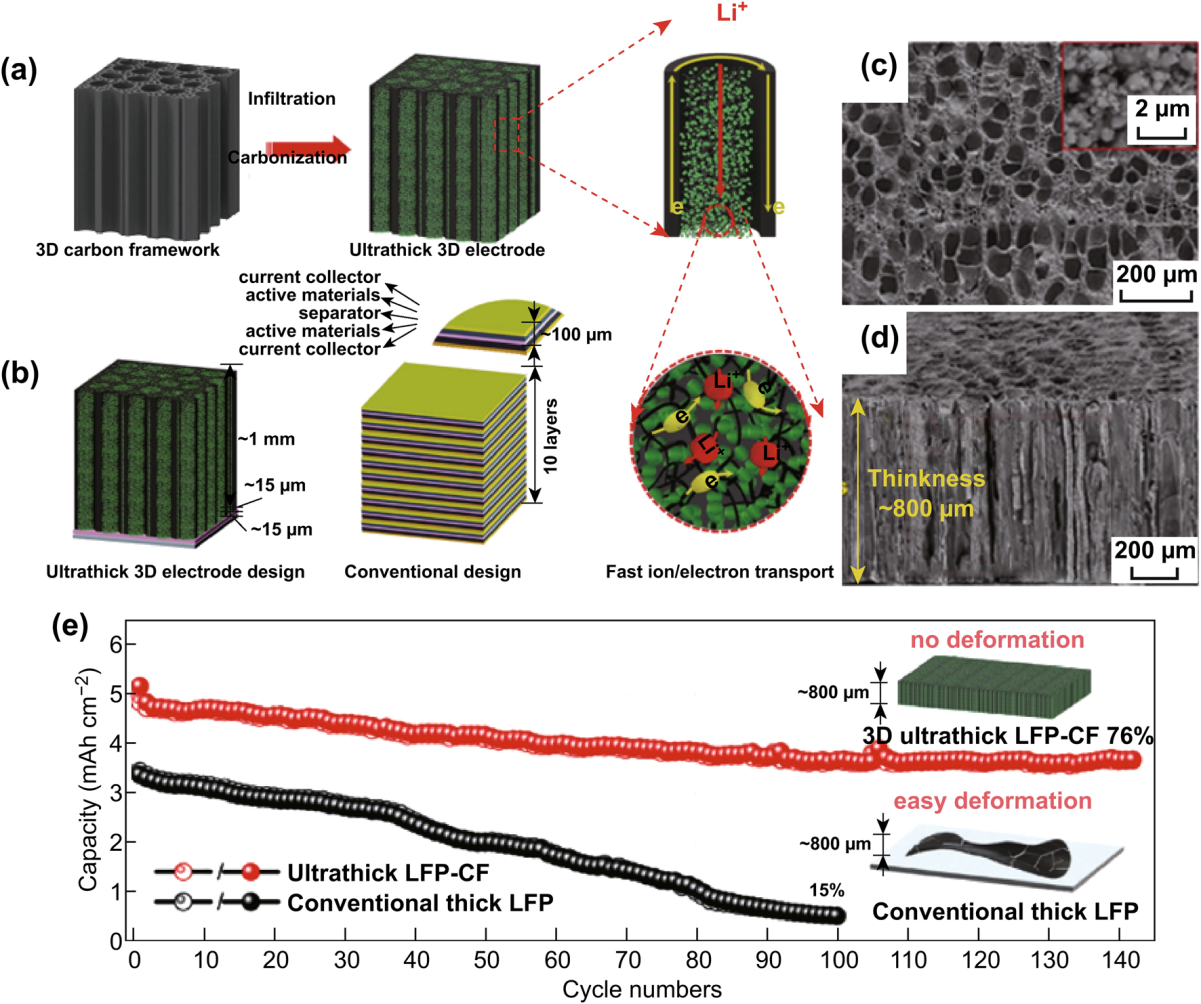
**a** Design concept of a 3D carbon-based current collector. **b** Visual comparisons of batteries with an ultrathick 3D electrode and a conventional design. Morphology and microstructure: **c** top view and** d **cross-sectional view. **e** Electrochemical performance. Reprinted with permission from Ref. [121]

Recently, 3D porous current collectors, instead of 2D planar current collectors, have attracted much attention for the preparation of free-standing and lightweight lithium-ion electrodes, such as CNT sponge-based 3D electrodes [116] and graphene foam (DGF)-based 3D electrodes [12, 117, 118]. As a typical example, using a 3D GF as both current collector and substrate for MoS_2_ growth, an integrated electrode of honeycomb-like-molybdenum sulfide@GF (HC-MoS_2_@GF) was fabricated [119]. Due to the direct contact and interconnection between the current and the active material, the HC-MoS_2_@GF exhibited high reversible capacity of 1235 mAh g^−1^ at 200 mA g^−1^ with excellent cycling stability. Inspired by the hierarchical porous structure of wood in nature [120], Chen et al. prepared a multi-channeled 3D carbon framework (CF) carbon-based current collector through the use of carbonized and activated multi-channeled natural wood (CA wood) (Fig. 8a, b) [121]. This 3D current collector displayed advantages of high electrical conductivity, low tortuosity, and lightweightness. By simple infiltration, the electroactive materials permeated into the channels of the 3D CF current collector to form an ultrahigh-mass-loaded and ultrathick (up to 800 µm) 3D electrode (Fig. 8c, d). Benefiting from its structural merits, this electrode exhibited significantly improved electronic and ionic kinetics; the ultrathick 3D electrode delivered a high capacity of 7.6 mAh cm^−2^ (95 Ah L^−1^ based on volume) at 0.5 mA cm^−2^, and an energy density of 26 mWh cm^−2^ (323 Wh L^−1^ based on volume) (Fig. 8e). Moreover, this 3D electrode exhibited excellent cyclability and overall safety in terms of its low deformability and enhanced mechanical properties. On the other hand, efforts have been made to fabricate 3D metallic current collectors (e.g., 3D Cu current collectors [122] or Ni foam [123]), which have the advantages of facile fabrication, large surface areas, and high porosities. In addition, 3D metallic collectors with uniform and smooth porous structures have been used in lithium-based batteries [124]. In comparison with 3D metallic current collectors, the lightweight carbonaceous current collectors still possess more reliable chemical stabilities and excellent pore controllabilities on the multiscale, which contribute to their better performance.

## Conclusions and Prospects

Engineering carbon electrodes is critical for fabricating both high energy density and power density LIBs. Nanostructured carbon-based electrodes hold great promise for enhancing electrochemical performance, which, unfortunately, is not readily scaled up to practical densities and thicknesses. Rationally assembled carbon architectures used in electrodes not only maintain the original nanoscale characteristics of each component, but also exploit synergism between different primary structures, thereby playing reinforcing roles in improving electrochemical performance under practical electrode conditions. As discussed in this review, a series of typical carbon architectures has been proposed to solve the problems of low electrical conductivities, sluggish ion kinetics, and large volume expansions in electrodes, yielding excellent cycling and rate performance (Table 1). For the carbonaceous active material, especially the conventional graphite anode, decreasing tortuosity in a directional assembly strategy to enhance the rate performance of a thick anode is highly effective. For the carbon framework design, a variety of effective carbon models in terms of interface, network, void space, and density have been well developed to solve structural and interfacial instabilities and enhance charge kinetics under practical levels of pressing density, mass loading, and discharging/charging rates in high-capacity noncarbon electrodes. Besides, strengthened interfacial interactions and well-designed ion diffusion pathways in carbon current collectors not only reduce the proportion of inactive materials in the whole device, but also enhance the energy and power densities of LIBs.

**Table 1:** Electrochemical performance of typical carbon architectures in lithium-ion batteries

Samples	Assembly approaches	Capacity (mAh g^−1^)	Cycle life (capacity retention%)	Rate performance (mAh g^−1^)	References
Si@void@C	Template and HF-etching	1160 mAh g^−1^ (0.5C)	1000	–	[30]
Si-NW/GNR paper	Vacuum-filtration	≈1500 mAh g^−1^ (1 A g^−1^)	300		[41]
LFP/GNR/G	Spray deposition and vacuum filtration	~130 mAh g_electrode_^−1^ (1C)	500	118 mAh g_electrode_^−1^ (5C)	[42]
Fe_3_O_4_/rGO paper	Magnetic field assisted self-assembly	1140 mAh g^−1^ (0.15 A g^−1^)	220	~700 mAh g^−1^ (10C)	[52]
VA-CNTs/Si	MPECVD and HF-CVD	800 mAh g^−1^ (10C)	100	765 mAh g^−1^ (15C)	[56]
Aligned graphite electrodes	Magnetic field assisted casting	~380 mAh g^−1^ (0.1 C)	−	~ 200 mAh g^−1^ (1C)	[62]
3D BNG	SiO_2_ template-assisted-CVD	725 mAh g^−1^ (1.5 A g^−1^)	200	51 mAh g^−1^ (2.09C)	[65]
GO/Sn_2_Fe-NRs array/rGO	Hydrothermal	90 mAh g^−1^ (0.5 A g^−1^)	600	682.5 mAh g^−1^ (2 A g^−1^)	[80]
MoS_2_@G	Electrospinning and CVD	1150 mAh g^−1^ (0.5 A g^−1^)	160	700 mAh g^−1^ (10 A g^−1^)	[87]
Gr-Si	CVD included CO_2_ oxidant	700 Wh L^−1^ (3.0 mAh cm^−2^)	200	2.7 mAh cm^−2^ (10C)	[88]
MoS_2_/VGNS	CVD and solvothermal	1109 mAh g^−1^ (0.2 A g^−1^)	100	818 mAh g^−1^ (2 A g^−1^)	[92]
RGO/TiO_2_(B) films	Grade layer-by-layer coating	128 mAh g^−1^ (6.7 A g^−1^)	~5000	74 mAh g^−1^ (20C)	[93]
Nb_2_O_5_/HGF	Self-assembly and H_2_O_2_ etching	~190 mAh g^−1^ (0.2 A g^−1^)	−	~80 mAh g^−1^ (20 A g^−1^)	[97]
Si/C microspheres	Ball milling, spray drying	620 mAh g^−1^ (0.1C)	500	~500 mA h g^−1^ (5C)	[98]
Yolk Sn@C nanobox	Solvothermal and annealing	810 mAh g^−1^ (0.2 A g^−1^)	500	350 mA h g^−1^ (4 A g^−1^)	[101]
Graphene-encapsulated SiMP	Sacrificial Ni conformal coating and CVD	1400 mAh g^−1^ (1.5 mA cm^−2^)	300	−	[102]
Ge-QD@ NG/NGF	Ni template-assisted CVD	1220 mAh g^−1^ (1C)	1000	801 mAh g^−1^ (40C)	[104]
GF/Si binder-free electrode	Freeze-drying	1170 mAh g^−1^ (1 A g^−1^)	1200	609 mAh g^−1^ (8 A g^−1^)	[105]
LiFePO_4_@C/rGO microspheres	One-pot mixed-solvothermal	112.4 mAh cm^−3^ (2C)	700	109.3 mAh g^−1^ (10C)	[109]
SnO_2_@GC	Sulfur-templated capillary shrinkage	974 mAh g^–^^1^/2123 mAh cm^−3^ (0.1 A g^−1^)	300	476 mAh g^−1^ (2 A g^−1^)	[113]
3D multi-channeled carbon-based current collector	Carbonization and activation	3.8 mAh cm^−2^ (2 mA cm^−2^)	140	1.7 mAh cm^−2^ (20 mA cm^−2^)	[121]

Although existing carbon engineering can promote lithium-ion-storage performance, various issues still remain in terms of practical applications, such as their high-costs, low coulombic efficiencies, and limited volumetric energy densities, especially for high-capacity and high-volume-change noncarbon anodes, which need to be solved in future lithium-ion-battery research. To achieve commercial production, the development of advanced carbon-based-material assembly strategies that involve inexpensive and simple process, including one-step methods, but are also highly efficient and easy to translate to industrial production, is extremely urgent. Promisingly, the design of carbon cages is potentially useful for application to high-capacity noncarbon anodes. As the most typical noncarbon anode material, the Si anode, with an ultrahigh theoretical capacity of 3590 mAh g^−1^, is at the advent of industrialization, but it suffers from the main challenge of huge volume expansion (above 300%) that leads to the continual loss of active material and the repetitive consumption of lithium resources (electrolyte and cathode). When the seriously unstable Si occupies a substantial portion of the electrode volume, carbon cages should be mechanically strengthened to house the volume-expanded silicon particles and block the intact electrolyte in order to protect the exposed surface during repeated lithiation. To simultaneously realize high volumetric performance and limited electrode expansion of the Si anode during battery charging, the carbon cages should be precisely designed to afford conformal compact protection with a trace amount. Therefore, the precise design of carbon cages for high-capacity noncarbon anodes is of great importance not only from the perspective of fundamental design studies into high volumetric materials, but also for promoting high-capacity noncarbon anodes based on nanocarbons into real electrochemical energy-storage devices.
